# Transmission Assessment Surveys (TAS) to Define Endpoints for Lymphatic Filariasis Mass Drug Administration: A Multicenter Evaluation

**DOI:** 10.1371/journal.pntd.0002584

**Published:** 2013-12-05

**Authors:** Brian K. Chu, Michael Deming, Nana-Kwadwo Biritwum, Windtaré R. Bougma, Améyo M. Dorkenoo, Maged El-Setouhy, Peter U. Fischer, Katherine Gass, Manuel Gonzalez de Peña, Leda Mercado-Hernandez, Dominique Kyelem, Patrick J. Lammie, Rebecca M. Flueckiger, Upendo J. Mwingira, Rahmah Noordin, Irene Offei Owusu, Eric A. Ottesen, Alexandre Pavluck, Nils Pilotte, Ramakrishna U. Rao, Dilhani Samarasekera, Mark A. Schmaedick, Sunil Settinayake, Paul E. Simonsen, Taniawati Supali, Fasihah Taleo, Melissa Torres, Gary J. Weil, Kimberly Y. Won

**Affiliations:** 1 Neglected Tropical Diseases Support Center, Task Force for Global Health, Decatur, Georgia, United States of America; 2 Division of Parasitic Diseases and Malaria, Centers for Disease Control and Prevention, Atlanta, Georgia, United States of America; 3 Department of Public Health, Ghana Health Service, Accra, Ghana; 4 Programme National d'Élimination de la Filariose Lymphatique, Ministère de la Santé, Ouagadougou, Burkina Faso; 5 Programme National d'Élimination de la Filariose Lymphatique, Ministère de la Santé, Lomé, Togo; 6 Department of Community, Environmental and Occupational Medicine, Ain Shams University, Cairo, Egypt; 7 Infectious Diseases Division, Washington University School of Medicine, St. Louis, Missouri, United States of America; 8 Centro National de Control de Enfermedades Tropicales, Santo Domingo, Dominican Republic; 9 Infectious Disease Office, National Center for Disease Prevention & Control, Manila, Philippines; 10 Neglected Tropical Diseases Control Programme, National institute for Medical Research, Dar es Salaam, Tanzania; 11 Institute for Research in Molecular Medicine, Universiti Sains Malaysia, Penang, Malaysia; 12 Epidemiology Department, Noguchi Memorial Institute for Medical Research, Legon, Ghana; 13 Department of Biological Sciences, Smith College, Northampton, Massachusetts, United States of America; 14 Department of Internal Medicine, Washington University School of Medicine, St. Louis, Missouri, United States of America; 15 Anti Filariasis Campaign, Ministry of Health, Colombo, Sri Lanka; 16 Division of Community and Natural Resources, American Samoa Community College, Pago Pago, American Samoa; 17 Faculty of Health and Medical Sciences, University of Copenhagen, Copenhagen, Denmark; 18 Department of Parasitology, University of Indonesia, Jakarta, Indonesia; 19 Neglected Tropical Diseases Unit, Public Health Directorate, Port Vila, Vanuatu; Michigan State University, United States of America

## Abstract

**Background:**

Lymphatic filariasis (LF) is targeted for global elimination through treatment of entire at-risk populations with repeated annual mass drug administration (MDA). Essential for program success is defining and confirming the appropriate endpoint for MDA when transmission is presumed to have reached a level low enough that it cannot be sustained even in the absence of drug intervention. Guidelines advanced by WHO call for a transmission assessment survey (TAS) to determine if MDA can be stopped within an LF evaluation unit (EU) after at least five effective rounds of annual treatment. To test the value and practicality of these guidelines, a multicenter operational research trial was undertaken in 11 countries covering various geographic and epidemiological settings.

**Methodology:**

The TAS was conducted twice in each EU with TAS-1 and TAS-2 approximately 24 months apart. Lot quality assurance sampling (LQAS) formed the basis of the TAS survey design but specific EU characteristics defined the survey site (school or community), eligible population (6–7 year olds or 1^st^–2^nd^ graders), survey type (systematic or cluster-sampling), target sample size, and critical cutoff (a statistically powered threshold below which transmission is expected to be no longer sustainable). The primary diagnostic tools were the immunochromatographic (ICT) test for *W. bancrofti* EUs and the BmR1 test (Brugia Rapid or PanLF) for *Brugia spp*. EUs.

**Principal Findings/Conclusions:**

In 10 of 11 EUs, the number of TAS-1 positive cases was below the critical cutoff, indicating that MDA could be stopped. The same results were found in the follow-up TAS-2, therefore, confirming the previous decision outcome. Sample sizes were highly sex and age-representative and closely matched the target value after factoring in estimates of non-participation. The TAS was determined to be a practical and effective evaluation tool for stopping MDA although its validity for longer-term post-MDA surveillance requires further investigation.

## Author Summary

Lymphatic filariasis (LF) is targeted for global elimination through a strategy of repeated annual mass drug administration (MDA) to entire at-risk populations. A transmission assessment survey (TAS) is designed to evaluate whether transmission of LF is presumed to have reached a level low enough that it cannot be sustained in the absence of drug intervention and, therefore, MDA can be stopped. This multicenter operational research trial examines the value and practicality of the TAS guidelines through its implementation in 11 countries of diverse geographical and epidemiologic profiles. The field experiences support the TAS survey design methodology with particular respect to school and cluster-based sampling strategies. We found that sample sizes were age and sex representative and met the target values after factoring in estimates of non-participation rates. In 10 of 11 countries, the TAS found the number of positive cases in the evaluation unit to be no more than the statistically powered critical threshold. These results were corroborated in a follow-up TAS approximately 24 months later. We conclude the TAS is a valuable and effective tool for stopping MDA but its utility for longer-term post-MDA surveillance needs further empirical evidence and may be best supported with complementary tools and methods.

## Introduction

Lymphatic filariasis (LF) is a mosquito-borne parasitic disease endemic to 73 countries worldwide. An estimated 1.4 billion people are said to be at-risk of LF disease with approximately 120 million infected and 40 million suffering from the crippling and stigmatizing clinical manifestations of the disease, especially lymphoedema and hydrocele [1]. As such, LF is one of the leading causes of chronic disability worldwide.

The primary focus for control and elimination of LF is the interruption of disease transmission through treatment of the entire at-risk population with repeated annual mass drug administration (MDA) using a single-dose combination of albendazole with either diethylcarbamazine (DEC) or ivermectin [2]. Since 2000, these efforts have been coordinated through the World Health Organization's (WHO) Global Programme to Eliminate Lymphatic Filariasis (GPELF), a collaborative public health program that has delivered to date nearly 4 billion drug treatments to over 950 million individuals in 53 countries [1]. This extraordinary achievement, made possible through the drug donations of manufacturers Merck (ivermectin) and GlaxoSmithKline (albendazole) has resulted in a marked reduction of infection prevalence in endemic areas, along with sizeable health and economic benefits to the affected populations [3], [4].

Essential to the Global Programme's success in combating LF is the important challenge of defining and confirming endpoints for MDA when disease transmission is presumed to have reached a level low enough that it cannot be sustained even in the absence of drug intervention. Given the biology and parasitic life cycle of LF, this threshold of infection is most likely to be reached following 4–6 annual MDA rounds with effective population coverage and a resulting microfilaria (mf) prevalence rate <1% (or Circulating Filarial Antigen [CFA] prevalence <2%) [2], [5]–[7]. The priority need, therefore, has been a standardized, robust evaluation tool to determine statistically whether this critical threshold has been met and recrudescence not likely to ensue if MDA is stopped. Earlier WHO guidelines for stopping MDA were notably cumbersome, resource-intensive, and conservative based on actual field implementation [8]–[10]. Because of these shortcomings, new guidelines were drafted [11] with the introduction of Transmission Assessment Surveys (TAS) that propose to be more logistically feasible and adaptable to varying demographic and epidemiologic conditions [12].

The present study was a multi-country operational research assessment of the TAS protocol, specifically aimed at evaluating the assumptions and accuracy of the TAS sampling strategy, as well as identifying best practices for TAS implementation. Results are informative for refining TAS methodologies and improving standard procedures going forward. Indeed, several of the preliminary results captured in this study were used by WHO to inform its development of the updated GPELF monitoring and evaluation guidelines [12].

## Methods

### Ethics Statement

Ethical approval was obtained for each participating country through the following review boards and institutions: Centers for Disease Control and Prevention Institutional Review Board (American Samoa), Comité d'ethique pour la Recherche en Santé du Ministère de la Santé (Burkina Faso), Consejo Nacional de Bioética en Salud (Dominican Republic), Noguchi Memorial Institute for Medical Research Institutional Review Board (Ghana), University of Indonesia Committee of the Medical Research Ethics (Indonesia), Ministry of Health Research and Ethics Committee (Malaysia), National Center for Disease Prevention and Control (Philippines), Ministry of Health (Sri Lanka), National Institute For Medical Research Clearance Committee (Tanzania), Comité Bioéthique de Recherche en Santé du Ministère de la Santé (Togo), Government of the Republic of Vanuatu Public Health Services (Vanuatu).

Informed assent was required from all sampled children in addition to written or oral consent from their parent or guardian. Oral consent was marked as part of the electronic record, witnessed by a teacher or other family member, and only used where allowed by the local ethical review board and not as a replacement for those countries requiring written consent (American Samoa, Malaysia, and Togo). Only data remaining in the country was identifiable; those electronically sent to the Task Force for Global Health were de-identified at the point of transmission. Positive children by any diagnostic test were treated with the appropriate medicines either during or immediately following the study. In Malaysia, however, only mf positive children were treated after the first TAS while all Brugia Rapid and mf positives were treated after the second TAS.

### Overview

For this study, eleven countries – American Samoa, Burkina Faso, Dominican Republic, Ghana, Indonesia, Malaysia, Philippines, Sri Lanka, Tanzania, Togo, and Vanuatu – took part in implementing and evaluating the TAS according to standard operating procedures (SOP) and guidelines [11], [12]. With the TAS applicable for both *stop-MDA* and *post-MDA surveillance* decision-making, countries were specifically chosen and sub-divided into these two categories based on their current MDA status. An initial TAS (referred to in this paper as TAS-1) was conducted over the period of September 2009 to February 2011. As prescribed by the TAS protocol, a second TAS (TAS-2) was done approximately two years later to re-evaluate the initial outcomes and decisions of TAS-1. All countries completed TAS-2 from September 2011 to April 2012 with the exception of American Samoa whose projected TAS-2 date extended beyond the timeline of this research study.

Within each country, TAS was carried out in a newly defined evaluation unit (EU) that was based on LF IUs used for MDA and follow-up assessments. An EU may consist of part of an IU, a whole IU, or a combination of multiple IUs that typically have contiguous borders and share similar epidemiologic profiles [12]. All EUs in this study met the TAS eligibility requirements of having completed at least five effective rounds of MDA in all IUs with coverage ≥65% of the total population, mf rates <1% (or CFA<2%) in all sentinel and spot-check sites post-5^th^ MDA, and a total population less than two million. In addition, at least six months had passed since the last MDA. Table 1 outlines each of the participating countries and corresponding EUs at the time of TAS-1.

**Table 1:** Evaluation Unit key characteristics at time of TAS-1.

TAS-1 Category	Country	TAS-1 Date	EU name	# of IUs in EU	# of MDAs	Last effective MDA	Total population	Area (km^2^)	Parasite species	Primary vector
**Stopping-MDA**	Burkina Faso	Dec-2009	Dafra-KV-Lena	3	7	2009	430,647	4,869	*W. bancrofti*	*Anopheles*
	Dominican Republic	Oct-2009	Southwest Focus	3	6	2008	158,672	1,882	*W. bancrofti*	*Culex*
	Ghana	Jan-2010	AES-Agona	2	8–9	2009	388,424	1,355	*W. bancrofti*	*Anopheles*
	Indonesia	Nov-2009	Alor	1	6	2007	178,964	3,012	*Brugia timori, W. bancrofti*	*Anopheles*
	Malaysia	Apr-2010	Sabah	9	5	2008	98,697	11,779	*Brugia malayi,*	*Mansonia*
	Tanzania	Nov-2009	Tandahimba	1	5	2007	240,121	2,260	*W. bancrofti*	*Anopheles*
**Periodic post-MDA surveillance**	American Samoa	Feb-2011	Tutuila	1	8	2009	65,000	151	*W. bancrofti*	*Aedes*
	Philippines	Oct-2009	Sorsogon	1	7	2007	681,840	2,188	*W. bancrofti*	*Aedes*
	Sri Lanka	Jan-2010	Dehiwala	<1	5	2006	230,518	22	*W. bancrofti*	*Culex*
	Togo	Dec-2009	Kozah	1	6	2008	229,798	1,103	*W. bancrofti*	*Anopheles*
	Vanuatu	Feb-2010	Penama	1	5	2004	26,646	1,305	*W. bancrofti*	*Anopheles*

### Survey Design

The TAS survey design depends upon factors such as the net primary school enrolment rate in each EU, the target population size, number of schools, vector type and parasite species to determine the required survey site, target population, survey type, sample size, critical cutoff, and diagnostic tool– all of which are described below and summarized in Table 2. A Microsoft Excel computer tool entitled *Survey Sample Builder* (SSB) was used to assist principal investigators (PI) in navigating the TAS protocol and inputting the required data [13]. From these inputs, the SSB produced random number lists and automated survey design calculations, including sample size and sampling intervals to facilitate rigorous sampling. In accordance with the TAS protocol, survey methodologies were identical for both stop-MDA and periodic post-MDA assessments.

**Table 2:** Survey design summary for TAS-1 and TAS-2.

Country	EU name	Survey site	Target population	Survey sample type	Target sample size	Critical cutoff	Primary Diagnostic tool
American Samoa	Tutuila	School	1^st^–2^nd^ graders	Systematic	1,042	6	ICT
Burkina Faso	Dafra-KV-Lena	Community	6–7 year olds	Cluster	1,556	18	ICT
Dominican Republic	Southwest Focus	Community	6–7 year olds	Cluster	1,532	18	ICT
Ghana	AES-Agona	School	1^st^–2^nd^ graders	Cluster	1,556	18	ICT
Indonesia	Alor	School	1^st^–2^nd^ graders	Cluster	1,548	18	PanLF, ICT (TAS-1) Brugia Rapid, ICT (TAS-2)
Malaysia	Sabah	School	1^st^–2^nd^ graders	Cluster	1,368	16	PanLF (TAS-1) Brugia Rapid (TAS-2)
Philippines	Sorsogon	School	1^st^–2^nd^ graders	Cluster	1,552	18	ICT
Sri Lanka	Dehiwala	School	1^st^–2^nd^ graders	Systematic	684	8	ICT
Tanzania	Tandahimba	Community	6–7 year olds	Cluster	1,540	18	ICT
Togo	Kozah	School	1^st^–2^nd^ graders	Cluster	1,548	18	ICT
Vanuatu	Penama	School	1^st^ graders	Census	933	.02N^1^	ICT

1 The critical cutoff in Vanuatu can be calculated exactly as .02N because the TAS was a census without random sampling error.

#### Survey site

School surveys were conducted in American Samoa, Ghana, Indonesia, Malaysia, Philippines, Sri Lanka, Togo, and Vanuatu, where net primary enrolment rates in the EU were ≥75%. In Burkina Faso and Tanzania where EU primary enrolment were <75%, community-based surveys were implemented using census enumeration areas (EA) and hamlets as the primary sampling units (i.e. clusters). Although school enrolment was ≥75% in the Dominican Republic, community-based surveys have commonly been used for LF evaluation here and were therefore preferred for the TAS with villages as the primary sampling unit. In this publication, the term EA will be used universally to designate primary sampling units for a TAS using community-based surveys.

#### Target population

Children 6–7 years old represent the target age group for TAS because they have lived most or all their lives during MDA and, therefore, positive filarial serology would be more indicative of *recent* LF transmission than it would be in older children or adults who may have been previously exposed. For school surveys, 1^st^ and 2^nd^ grade children were chosen as a proxy for 6–7 year olds; all children in these grades were eligible including those outside this age range. In Togo, however, only 6–7 year old children in 1^st^ and 2^nd^ grade were surveyed. Another exception was in Vanuatu where only 1^st^ grade children were enrolled due to the expected large proportion of children aged nine and above in Grade 2. Community surveys specifically targeted and sampled 6–7 year old children only.

#### Survey type

For each country, it was determined whether a *cluster* or *systematic* survey was most appropriate. *Cluster*-*sample* surveys were used in Burkina Faso, Dominican Republic, Ghana, Indonesia, Malaysia, Philippines, Tanzania, and Togo where EUs had a sampling frame of at least forty primary sampling units and a large number of target eligible children. In these countries, only a subset (≥30) of total schools or EAs was randomly selected for sampling. For EUs in American Samoa and Sri Lanka with smaller target populations and fewer primary sampling units, the TAS required *systematic* sampling rather than cluster sampling. In Vanuatu where the target population was very small, the selection method was a *census* where all primary schools and eligible children in the EU were surveyed.

#### Sample size and selection

The survey type, target population size, and LF vector in the EU determined the target sample size for each country. All else being equal, TAS sample sizes were larger in EUs where cluster surveys were required. The TAS assumes a cluster survey design effect of 1.5 if the target population is <2400 (<5000 for *Aedes* EUs) and 2.0 if ≥2400 (≥5000 for *Aedes* EUs); these initial estimates were based on low expected prevalence of filarial antigenaemia following several MDAs and, therefore, lower probability of prominent clustering. TAS sample sizes are also larger where *Aedes* is the primary LF vector because it is known to be more efficient at transmitting the infection. Target sample sizes in this study were provided by SSB and ranged from 684 in Sri Lanka (systematic sampling) to 1556 in Burkina Faso and Ghana (cluster sampling).

The selection of clusters was done by systematic sampling without regard to school or EA size. Schools or EAs were listed in order of proximity from a central point to ensure a geographically representative sample across the entire EU. A random starting site was selected by SSB, based on a random number chosen between one and the sampling interval, and the calculated sampling interval then applied to determine the remaining survey sites. If the target sample size was not met after all clusters were surveyed, an additional set of at least 5 clusters was randomly selected from the remaining pool without replacement.

The same systematic sampling approach was used in cluster-sample surveys to select children within each school or EA in Burkina Faso, Ghana, Indonesia, Malaysia, and Togo, where average cluster size was large enough that only a fraction of eligible children were needed at each site to reach the required sample size.

#### Critical cutoff

The TAS critical cutoff value represents the threshold of infection prevalence below which transmission is expected to be no longer sustainable, even in the absence of MDA. TAS estimates the EU's relationship to this threshold by the number of serologic antigen- or antibody-positive cases. If the total number of positive cases is at or below the critical cutoff, the EU ‘passes’ the survey and the decision to stop MDA can be made. If the total number of positive cases is above the critical cutoff, MDA should continue in the EU for at least two more rounds [12].

TAS sample sizes and critical cutoff values are powered so that the EU has at least a 75% chance of passing if the true antigen or antibody prevalence is half the threshold level (2% for *Culex, Anopheles*, and *Mansonia* vector areas, and 1% for *Aedes* vector areas). In addition, there is no more than a 5% chance of passing if the true prevalence is greater than or equal to the threshold level.

#### Diagnostic tools

Following a comprehensive multicenter study evaluating potential diagnostic tools [14], the immunochromatographic (ICT) test for filarial antigen and the BmR1 antibody test (PanLF or Brugia Rapid) were recommended for TAS in *W. bancrofti* and *Brugia spp.* endemic areas, respectively. These were, therefore, the principal diagnostic tools used for evaluation in this study, in accord with test-specific SOPs and the following procedures:

- For TAS-1, positive ICT tests were immediately followed up with a second confirmatory ICT test. If the second test was also positive, the child was considered positive; however, if the second test was negative, the child was considered negative. Because of satisfactory reproducibility results in TAS-1, the repeat of positive ICT tests was dropped in TAS-2.
- For TAS-1, the PanLF test was used in *Brugia spp.* countries (i.e. Indonesia, Malaysia). For TAS-2, the Brugia Rapid test was used due to wider availability; however, both tests incorporate the same BmR1 recombinant filarial antigen to measure antibodies and proved to have similar sensitivity in multicenter evaluations [15].
- With the EU in Indonesia endemic for both *W. bancrofti* and *Brugia spp.*, ICT and PanLF tests were individually conducted for all sampled children in TAS-1. For TAS-2, however, only Brugia Rapid tests were used; ICT tests have yet to be completed because of logistic complications importing the cards into the country.
- Positive ICT or PanLF or Brugia Rapid children were followed up with mf testing. The three-line blood smear technique was conducted in TAS-1 and TAS-2 in local in-country laboratories [14]. In Tanzania, the counting chamber technique was used for the detection of mf in place of the three-line blood smear. Additional mf testing using the real-time polymerase chain reaction (PCR) procedure [14] was performed at Smith College (Northampton, USA) but only for TAS-1 in all countries.

### Data Collection

For TAS-1, survey data were collected on personal digital assistants (PDA) (Hewlett Packard iPAQ 211) using the Electronic Data Gathering and Evaluation (EDGE) system designed at the Task Force for Global Health (Decatur, USA) [14]. Each child was assigned a unique identification number that was printed on a barcode label, scanned into the PDA with a Bluetooth scanner (Socket Mobile CX2821-656), and affixed to blood vials and diagnostic tests to facilitate specimen management. External global positioning system (GPS) cards (GlobalSat BC-337) were attached to the PDA to track GPS coordinates of each school or household surveyed. The survey itself consisted of questions capturing basic location, demographic, and test result information. Each night, the PDA was synchronized to a central laptop and the data were sent to a secure server at the Task Force for Global Health whenever the Internet was next accessible.

For TAS-2, identical survey data were collected but on mobile smartphones (Motorola Milestone XT720) through a modified version of the *OpenDataKit* (ODK) application developed at the University of Washington (Seattle, USA). The same barcode identification and labeling procedure as TAS-1 was implemented using the *Barcode Scanner* application (Zxing Team). All phones were equipped with a built-in GPS. Collected data were automatically sent through cellular service or wireless Internet to a secure server at the Task Force for Global Health.

### Field Activities

Each country used 3–5 field teams consisting of at least three persons each: a data collector, phlebotomist, and supply manager. All team members and supporting staff were trained on the study SOPs and electronic data collection tools by external consultants.

For school surveys, field teams were assisted by teachers who provided official class registers and identified all survey-eligible children for enumeration and selection according to the SSB randomized number lists. For each selected child, demographic data (i.e. name, sex, age) were recorded and blood collected (at least 100 µl for ICT, 60 µl for PanLF, and 35 µl Brugia Rapid tests) via finger prick into an EDTA-coated tube [14]. The single exception was in Vanuatu TAS-1 where blood was collected into a calibrated capillary tube and directly applied onto the ICT card for reading at the school due to logistic challenges.

For community-based surveys, a household selection process was required in the selected EAs. With the assistance of local officials and sketch maps, field teams identified a route through the EA passing and enumerating each house. All houses corresponding to the SSB randomized number lists were selected and all 6–7 year olds residing at the chosen households were surveyed with the requisite blood collected in anticoagulant tubes. One exception to this procedure occurred in Tanzania where instead of going house to house, hamlet leaders organized all 6–7 year olds at a central location in advance of the survey team's arrival for selection and sampling. The small population and area of the hamlets, in addition to accurate updated registries and strong working relations between health staff and hamlet leaders, permitted this strategy and mitigated concerns about potential selection bias. In contrast, this approach was not operationally feasible in Burkina Faso because of larger EA sizes including peri-urban areas, as well as sampling intervals >1 so household enumeration was necessary.

Following specimen collection, all blood-filled tubes were stored and transported via cold-chain to a nearby laboratory base to process and record ICT (or PanLF, Brugia Rapid) test results. Children testing positive were identified using the EDGE/ODK systems and individually followed up at night during peak mf hours to collect an additional blood sample for mf testing (10pm–2am except in American Samoa where *W. bancrofti* shows diurnal periodicity).

The number of children absent on the survey date was recorded for all surveys. For community surveys, field teams made at least one revisit to the absent child's house before recording an official absence. The number of selected children without consent or refusing to participate was also captured in addition to invalid and incomplete tests due to malfunction or insufficient blood. Together, these absentees, refusals, and individuals with test errors were designated as TAS *non-participators*.

### Data Analysis

All transmitted data were compiled into a central database at the Task Force for Global Health and exported into Microsoft Excel spreadsheets for final cleaning and approval by the collaborating principal investigators. Statistical analysis was done by importing the clean datasets into SAS v9.3 (SAS Institute). Summary statistics of test results and univariate analyses with regard to age, sex, and location were performed using the PROC UNIVARIATE function. Design effect calculations were conducted using the PROC SURVEYFREQ function.

## Results

### TAS Results

*For W. bancrofti countries*, TAS-1 and TAS-2 results are presented in Table 3. All EUs passed TAS-1, meaning that the number of ICT positive children was no greater than the critical cutoff value. As recommended by the TAS, MDA was then stopped (or periodic post-MDA surveillance continued) in those specific EUs for approximately 24 months before conducting TAS-2. All *W. bancrofti* EUs (with the exception of American Samoa and Indonesia where follow-up assessments were not yet completed) also passed TAS-2, thereby corroborating the TAS-1 stop-MDA or post-MDA surveillance decision. Microfilaraemia (mf) tests were conducted on ICT positive children using the three-line blood smear (TAS-1 and TAS-2) and PCR (TAS-1) procedures. The proportion of mf-positive children among antigen-positive children identified in the TAS was low in the *W. bancrofti* countries. The positive blood smear to positive ICT proportion was 12.9% (4/31) for TAS-1 and 5.2% (1/19) for TAS-2, while the proportion of positive PCR to positive ICT was 22.6% (7/31).

**Table 3:** ICT, blood smear, and PCR results for *W. bancrofti* countries.

	ICT (Ag)			Blood smear (mf)		PCR (mf)
Country	Critical cutoff value	TAS-1 positive^1^	TAS-2 positive^1^	TAS-1 positive^2^	TAS-2 positive^2^	TAS-1 positive^2^
Am. Samoa^3^	6	2/949	n/a	0/2 (0.0%)	n/a	0/2 (0.0%)
Burkina Faso	18	13/1571	5/1591	2/13 (15.4%)	0/5 (0.0%)	5/13 (38.5%)
Dom. Rep.	18	0/1609	3/1558	-	1/3 (33.3%)	-
Ghana	18	2/1557	0/1514	0/2 (0.0%)	-	0/2 (0.0%)
Indonesia^4^	18	6/1312	n/a^4^	0/6 (0.0%)	n/a^4^	0/6 (0.0%)
Philippines	18	2/1599	1/1656	0/2 (0.0%)	0/1 (0.0%)	0/2 (0.0%)
Sri Lanka^3^	8	0/679	1/698	-	0/1 (0.0%)	-
Togo	18	2/1571	0/1550	1/2 (50.0%)	-	1/2 (50.0%)
Tanzania	18	10/1561	9/1588	1/10 (10.0%)	0/9 (0.0%)	1/9 (11.1%)
Vanuatu	18, 19^5^	0/933	2/954	-	0/2 (0.0%)	-

1 % of total survey population.

2 % of ICT+ individuals; some individuals could not be retraced for mf testing.

3 Systematic sampling was used in American Samoa and Sri Lanka.

4 Indonesia EU of Alor+Pantar islands is endemic for both *W. bancrofti* and *Brugia timori*. TAS-2 ICT tests were not available due to logistic problems importing diagnostic tests into the country.

5 Census critical cutoff value is equal to .02N for EUs with *Culex*, *Anopheles*, or *Mansonia* as primary LF vector.

*For Brugia spp. countries,* Indonesia passed TAS-1 and TAS-2 and only one mf positive was found across both surveys (Table 4). The number of PanLF positive children in Malaysia (Sabah), however, exceeded the critical cutoff value in TAS-1. MDA was, therefore, continued before re-testing in TAS-2, but for only one round in 8 IUs due to DEC supply problems. Results for TAS-2 using the Brugia Rapid test were still greater than the critical cutoff value so consequently, MDA has been recommended to continue in the EU for two more rounds before conducting another TAS evaluation. Mf results in Malaysia (Sabah, not peninsular Malaysia) confirmed a high likelihood of active transmission with a TAS-1 positive blood smear to positive PanLF proportion of 35.6% (32/90) and positive PCR to positive PanLF proportion of 52.2% (47/90). The TAS-2 positive blood smear to positive Brugia Rapid proportion decreased to 20.5% (15/73) following the additional rounds of MDA.

**Table 4:** PanLF, Brugia Rapid, blood smear, and PCR results for *Brugia spp.* countries.

	PanLF or Brugia Rapid (Ab)			Blood smear (mf)		PCR (mf)
Country	Critical cutoff value	TAS-1 (PanLF) positive^1^	TAS-2 (Brugia Rapid) positive^1^	TAS-1 positive^2^	TAS-2 positive^2^	TAS-1 positive^2^
Indonesia^3^	18	12/1353	14/1622	0/12 (0.0%)	1/14 (7.1%)	0/12 (0.0%)
Malaysia	16	90/1429	73/1684	31/87 (35.6%)	15/73 (20.5%)	46/86 (53.4%)

1 % of total survey population.

2 % of PanLF(+) or Brugia Rapid(+) individuals; some individuals could not be retraced for mf testing.

3 Indonesia EU of Alor and Pantar islands is endemic for both *W. bancrofti* and *Brugia timori*.

### Population and Sampling Characteristics

The proportions of male and female children sampled were very even across all school and community-based surveys in both TAS-1 and TAS-2 (Table 5). In addition, no one country in either survey had more than 54% male or female children in the sample.

**Table 5:** TAS sample size by sex for school and community-based surveys.

Sex	School TAS (16 surveys)	Community-based TAS (6 surveys)	Total (22 surveys)
Male	9,894 (50.2%)	4,752 (50.1%)	14,646 (50.2%)
Female	9,798 (49.8%)	4,725 (49.9%)	14,523 (49.8%)
Total^1^	**19,692 (100.0%)**	**9,477 (100.0%)**	**29,169 (100.0%)**

1 *57 records were missing sex identification data.*

The target age group for TAS is 6 and 7 year old children, approximated by 1^st^ and 2^nd^ graders in school surveys. In *W. bancrofti* EUs, 84% of the total sample in school surveys was aged 6 and 7 and 95% between 6 and 10 years old (Table 6). The *Brugia spp.* EUs in Indonesia and Malaysia found a higher proportion of 8 year olds in the TAS sample due to 1^st^ and 2^nd^ grade in both countries primarily consisting of 7 and 8 year old children. No positive cases were detected outside the 6–10 year old range although one positive ICT test was associated with a child of unspecified age.

**Table 6:** TAS results by age for school surveys in *W. bancrofti* and *Brugia. spp.* countries.

	*W. bancrofti* countries^1^		*Brugia spp.* countries	
Age (years)	n (% of total)	ICT+ (% of age)	n (% of total)	PanLF or Brugia Rapid+ (% of age)
<6	694 (4.7%)	0 (0.0%)	160 (2.6%)	2 (1.3%)
6–7	12,479 (83.7%)	11 (0.1%)	2,713 (44.5%)	79 (2.9%)
8–10	1,689 (11.3%)	6 (0.4%)	3,213 (52.8%)	108 (3.4%)
>10	37 (0.3%)	0 (0.0%)	2 (0.1%)	0 (0.0%)
Total^2^	**14,899 (100.0%)**	**17 (0.1%)**	**6,088 (100.0%)**	**189 (3.1%)**

1 Includes TAS-1 ICT tests for Indonesia.

2 *73 records were missing age data (including 1 ICT+).*

Table 7 is informative because it displays the target and actual sample sizes for TAS-1 and TAS-2 along with the number of clusters (schools or EAs) surveyed to achieve the total. The target sample size was mostly met in both surveys with a few notable exceptions. In American Samoa TAS-1, there was insufficient blood to perform the ICT test in a number of collected samples. Likewise in Indonesia TAS-1, ICT and PanLF tests were unavailable at the time of sampling; therefore, they were conducted retroactively using preserved serum and several samples did not have enough quantity to complete the test. For TAS-2 in Malaysia, the actual sample size greatly exceeded the target due to the random selection of several large schools in addition to a lower non-participation rate than initially estimated.

**Table 7:** Comparison of target and actual sample sizes and number of clusters.

Country	Survey	Target sample	Actual sample^1^	% difference	Original clusters selected	Extra clusters needed
Am. Samoa	TAS-1	1,042	949	−8.9%	26^2^	-
	TAS-2	-	-	-	-	-
Burkina Faso	TAS-1	1,556	1,571	1.0%	30	1
	TAS-2	1,556	1,591	2.2%	30	8
Dom. Rep.	TAS-1	1,532	1,609	5.0%	30	8
	TAS-2	1,532	1,558	1.7%	40	0
Ghana	TAS-1	1,556	1,557	0.1%	30	10
	TAS-2	1,556	1,514	−2.7%	30	2
Indonesia	TAS-1	1,548	1,353	−12.6%	30	13
	TAS-2	1,548	1,622	4.8%	30	0
Malaysia	TAS-1	1,368	1,429	4.5%	30	2
	TAS-2	1,368	1,684	23.1%	33	0
Philippines	TAS-1	1,552	1,599	3.0%	35	10
	TAS-2	1,552	1,656	6.7%	35	0
Sri Lanka	TAS-1	684	679	−0.7%	35^2^	-
	TAS-2	684	698	2.0%	32^2^	0
Togo	TAS-1	1,548	1,571	1.5%	30	1
	TAS-2	1,540	1,550	0.6%	39	0
Tanzania	TAS-1	1,540	1,561	1.4%	51	18
	TAS-2	1,540	1,588	3.1%	70	0
Vanuatu	TAS-1	933	933	0.0%	63	0
	TAS-2	954	954	0.0%	63	0
**Total**	**TAS-1**	**14,859**	**14,811**	**−0.3%**	**390**	**63**
	**TAS-2**	**13,830**	**14,415**	**4.2%**	**402**	**10**

1 *Excluding invalid tests and specimens unable to be tested.*

2 *Systematic sampling; all eligible primary sampling units surveyed.*

Table 7 also presents the number of original clusters selected and the number of extra clusters needed to meet the sampling requirements. In TAS-1, a total of 63 extra clusters were required, most prominently in Ghana, Indonesia, Philippines, and Tanzania. In contrast, only 10 total extra clusters were required in TAS-2, primarily as a result of factoring the non- participation rates into the SSB survey design calculation. The non- participation rate includes children – enrolled in first and second grade (for school surveys) or residing in the selected house (for community-based surveys) – absent on the survey date and those refusing to participate or without consent. The rate was a combined 14.0% for TAS-1 and 10.2% for TAS-2 but varied by country and survey (Table 8). Non-participators also include invalid (i.e. malfunctioning) diagnostic tests or samples that were collected but had insufficient quantity or other barriers preventing completion of the test (e.g. blood clotting). These specific non- participation factors accounted for approximately 4% of total TAS-1 and 2% of total TAS-2 samples but were also dependent on country and survey. Some non- participation rates were not tracked or estimated in American Samoa (TAS-1), Burkina Faso (TAS-1), and Sri Lanka (TAS-1).

**Table 8:** Non-participation rates observed in TAS-1 and TAS-2.

		Absent, refused, or no consent		Invalid test or Unable to be tested	
Country	Survey site	TAS-1	TAS-2	TAS-1	TAS-2
Am. Samoa	School	-	-	16.0%	-
Burkina Faso	Community	-	7.5%	0.9%	0.3%
Dom. Rep.	Community	12.6%	7.2%	0.6%	0.1%
Ghana	School	15.0%	15.0%	0.1%	2.9%
Indonesia	School	20.0%	10.0%	18.3%	9.5%
Malaysia	School	22.9%	20.4%	0.3%	0.5%
Philippines	School	4.0%	3.0%	4.0%	1.3%
Sri Lanka	School	-	9.3%	0.0%	1.4%
Togo	School	12.0%	8.0%	0.0%	0.0%
Tanzania	Community	14.7%	5.7%	0.6%	1.1%
Vanuatu	School	10.7%	15.7%	0.0%	0.0%
Total	**-**	**14.0%**	**10.2%**	**3.8%**	**1.9%**

Design effects for TAS-1 and TAS-2 cluster surveys are listed in Table 9. All *W. bancrofti* countries had design effects less than the TAS estimated value of 2 (for target populations >2400), indicating the required sample size was not underestimated. Conversely, Indonesia and Malaysia, both *Brugia spp.* EUs, had design effects larger than 2 that may be associated with the more sensitive detection of antibody versus antigenemia, and with the subsequently larger number of positive cases found, particularly in Malaysia.

**Table 9:** Design effects calculated for TAS-1 and TAS-2 cluster surveys.

Country	TAS-1	TAS-2
Burkina Faso	1.3	0.8
Dom. Rep.	-	1.6
Ghana	2.0	-
Indonesia	2.5	2.2
Malaysia	7.9	7.0
Philippines	1.0	1.0
Togo	0.9	-
Tanzania	1.1	1.1

### Time and Costs of These Studies

The overall average number of field days required for TAS was 26 in TAS-1 (range: 9–60) and 27 for TAS-2 (range: 12–50), using an average number of 4 field teams (range: 3–6) with 3–4 persons per team (Table 10). School surveys took 24–27 days on average versus 26–33 for community surveys but the overall survey length was highly dependent on country-specific factors including weather, distance, and other logistic delays, particularly in the Philippines, Dominican Republic, Indonesia, and Vanuatu.

**Table 10:** Number of field days required to complete TAS-1 and TAS-2.

Survey site	Country	Field days TAS-1	Field days TAS-2	Field teams TAS-1 and TAS-2
School	Am. Samoa	9	-	6
	Ghana	20	18	4
	Indonesia	35	18	6
	Malaysia	18	18	5
	Philippines	60	50	3
	Sri Lanka	26	32	3
	Togo	14	12	3
	Vanuatu	25	25	4
	***Average***	***27***	***24***	***4***
Community	Burkina Faso	19	18	3
	Dom. Rep.	57	42	3
	Tanzania	22	19	3
	***Average***	***33***	***26***	***3***
All sites	***Average***	**26**	**27**	**4**

The mean and median TAS costs in this operational research study were $25,500 and $24,900 with the largest proportion of costs allocated to personnel (33%) and transportation (24%) (Tables 11 and 12). Community surveys (mean $26,800, median $26,000) required slightly more resources than school surveys (average $24,900, median $23,800). Project cost was moderately correlated to the area of the EU (R^2^ = .39). It should be noted, however, that all costs referenced here reflect research budgets and objectives including training, foreign consultants, and extra specimen shipment and analysis; carried out for programmatic purposes, costs would be expected to be less.

**Table 11:** Total TAS operational research costs for school and community-based surveys.

Survey site	Low	High	Mean	Median
School (n = 8)	$16,200	$36,900	$24,900	$23,800
Community (n = 3)	$17,500	$36,800	$26,800	$26,000
Total (n = 11)	**$16,200**	**$36,900**	**$25,500**	**$24,900**

**Table 12:** Allocation of TAS costs by spending category.

Description	% of total costs
Personnel (per diems)	33%
Transportation (fuel, vehicle hire)	24%
Diagnostic tests (procurement, shipment, customs)	15%
Consumable supplies (e.g. lancets, EDTA tubes)	14%
Communication (e.g. printing, mobile phone data)	3%
Other (e.g. training, consultants, sensitization, specimen shipment)	11%
Total	**100%**

## Discussion

LF elimination programs require a standardized methodology that is statistically robust and programmatically feasible in order to assure confidence in making stop-MDA and post-MDA surveillance decisions. In this regard, Transmission Assessment Surveys offer a more pragmatic approach than previous WHO guidelines and with 22 implementations of the TAS in 11 countries, this operational research study provides the first report of a large-scale rollout of the TAS at a programmatic level. Indeed, these field experiences in multiple geographic and epidemiological settings have offered a prime opportunity to evaluate the TAS protocol critically and identify both best practices for future implementation and important remaining research gaps.

### TAS Results and Sampling Strategy

Consistent results were seen across TAS-1 and TAS-2. In the 10 EUs that passed TAS-1, the recommended decision to stop MDA was validated in TAS-2, as no resurgence of infection was observed above the critical cutoff value where active transmission is anticipated as likely to occur. This finding is extremely important from a programmatic perspective because if the TAS-2 result had differed from TAS-1, MDA might have needed to be restarted in the EU, which is not only a resource intensive process but one that could be politically and socially undesirable. A final TAS evaluation is recommended in these EUs after another 2–3 years to confirm the absence of reemerging transmission detectable by the TAS.

The results were in-line with anticipated outcomes of the TAS survey design and sampling strategy. Design effects for *W. bancrofti* EUs fell within expected limits, and participant age and sex reflected distributions in the target population. One notable advantage of the TAS protocol is its inclusion of cluster surveys to reduce the number of survey sites and overall sample size. In this study, 8 of 11 countries used a cluster survey design although sampling efficiency differed from TAS-1 to TAS-2. For TAS-1, a total of 63 extra clusters had to be selected and surveyed in addition to the originally planned sample in order to fulfill the target sample size. Such a process proved burdensome to survey planning and resource allotment. In contrast, only 10 extra clusters were needed in TAS-2 to achieve the target objective. This vast improvement in TAS-2 is largely because of factoring in ‘non-participators’ (i.e. absent children and those refusing to participate or without consent) into the initial survey design calculation. Estimates of the non- participation rate, however, might be difficult to obtain or measure during TAS planning, as was the experience in several of the countries in TAS-2. In such cases, a 10–15% estimated non- participation rate can be recommended based on the results from this study (Table 9), although this rate may vary greatly by EU and survey location. Community-based surveys, in particular, may experience a larger non- participation rate than school surveys because of the unreliable availability of eligible children at specific times of the day. The amount of TAS pre-planning and school or community sensitization is also likely to influence non- participation rates considerably. Because the TAS uses a fixed sampling fraction within each cluster, the inclusion of an accurate non- participation rate into the survey design calculation is also necessary to achieve a more accurate sample size. More specifically, underestimating the non- participation rate would result in larger sampling intervals and, therefore, fewer children sampled per cluster than required given the number of clusters selected. Since the TAS presumes an equal probability sample, extra clusters would be needed to make up the sample size difference, as seen most notably in TAS-1.

Despite best efforts to reach sample size targets efficiently using non- participation rates and extra clusters, our study found that discrepancies may persist because of outdated population or enrollment estimates, school closures, inclement weather, and other factors including the selection by chance of several large or small outlier schools. Non-participation is also not unprecedented in such types of surveys and because absentees were randomly spread out across clusters, sampling bias was likely not introduced. Furthermore, the inclusion of extra clusters improved sample robustness and reduced intraclass correlation between clusters. Probability proportional to estimated size (PPES) sampling has been investigated but preliminary assessment suggests the uncertainties of actual school size and number of smaller schools with target children below the fixed number needed would increase the average clusters required and likely offset benefits to standardizing the sample size [16]. Strategic approaches to harmonize the target and actual sample size will likely evolve as the TAS is further field tested and evaluated. Several improvements have already been made to the SSB tool including the input of an estimated non- participation rate and the automatic random selection of ‘backup clusters’ to survey in case the target sample size is not initially met. This study also validated the overall utility and convenience of the SSB tool with regards to simply determining the proper survey design, calculating sample sizes and sampling intervals, and randomizing cluster and child selection lists. Future TAS should continue using the SSB tool for survey planning.

The TAS protocol identifies 6–7 year old children as the target age group. While no positive cases were found outside the 6–10 year age range, a narrower sampling frame of 6–7 year olds is believed to be more epidemiologically accurate and programmatically feasible to avoid larger sample sizes [16]. In school surveys, 6–7 year olds are approximated by 1^st^–2^nd^ grade children. This approximation, however, proved ambiguous in countries where the target ages and grades did not effectively align. For example, in Ghana, children 8–10 years were frequent in 1^st^–2^nd^ grade. In Malaysia and Indonesia, 1^st^–2^nd^ grade typically corresponds to 7–8 year old children. Furthermore, some countries including Togo interpreted the guidelines as only including 6–7 year olds within 1^st^–2^nd^ grade as the target population. Therefore, although the results show that 6–7 year old children still comprised the majority of all school surveys, the clarification of the age requirement in the TAS protocol is extremely important for planning and calculating an accurate survey design. To this end, the general guideline in the SSB tool has been revised for programs to specifically select the grade(s) in which 6–7 year old children are most likely to be found and then to use those grade(s) as the eligible target group for school surveys. This refined terminology was implemented successfully in the Vanuatu study and is likely to benefit and simplify future TAS implementations as well.

### Specimen Collection and Diagnostic Tests

Specimen collection procedures were closely examined within the context of an operational research protocol that involved collecting blood into an EDTA-coated tube that would be transported and analyzed in a central laboratory, as opposed to directly conducting the ICT (or PanLF, Brugia Rapid) tests in the field. The perceived advantage of this method was to streamline blood collection in the field while being able to perform the diagnostic tests in a more controlled environment. This strategy proved adequate under operational research conditions to evaluate quality and consistency; however, it introduced logistic challenges in terms of transportation, time, and supplies. In addition, it was observed that field staff may be unfamiliar with drawing blood into EDTA tubes and basic pipetting techniques. This method was also more challenging for follow-up testing or where there was insufficient blood quantity or clotting. As a result, it may be more efficient programmatically for teams to conduct diagnostic tests in the field, directly transferring blood from the finger prick to the ICT or Brugia Rapid card with a calibrated capillary tube. This process was carried out successfully in Vanuatu, Indonesia, and Malaysia because of logistic restrictions that are likely to be duplicated in other TAS-eligible EUs. However, because the rapid diagnostic tests are extremely time sensitive and require good lighting, it is highly recommended that one team member be specifically assigned to timing and reading the tests in an area with sufficient lighting. However, in community surveys where house-to-house visits are more time consuming and on-the-spot diagnostic testing is likely to exacerbate this constraint, especially when surveys are conducted in the afternoon or evening, lighting becomes more restricted and it might be preferable to collect blood in EDTA tubes for later analysis.

The performance and reliability of the diagnostic tests used for the TAS are undoubtedly critical to the success of the survey. In TAS-1, all positive ICT tests were immediately followed-up with a repeat test to confirm the initial finding. In all 33 positive cases, the original and repeat ICT tests were both positive, indicating 100% positive concordance. Despite this limited sample size, repeat ICT tests are deemed unnecessary under current TAS programmatic guidelines. More importantly, however, the field experiences here showed that the quality and consistency of ICT results can be strongly improved with robust training and strict adherence to reading the cards after exactly ten minutes. A newer filariasis test strip with potential greater sensitivity and reduced susceptibility to heat will only improve the accuracy of TAS results although it may require the adjustment of critical cutoff values and sample sizes [17].

Mf tests using blood smear (TAS-1 and TAS-2) and PCR methods (TAS-1 only) were examined in this study and showed that positive concordance to antigen (*W. bancrofti*) and antibody (*Brugia spp.*) results were comparable to previous studies, albeit with much smaller sample sizes [14]. Programmatically, however, the ICT and Brugia Rapid tests remain more suitable as the primary TAS diagnostic tool given their convenience advantages. Mf tests may best be utilized as a positive-case follow-up tool to test for potential hotspots, focal transmission, or spatial clustering.

### School versus Community-Based TAS in Targeted EUs

The community-based TAS studies in Burkina Faso and Tanzania highlighted several specific challenges; in particular, both had trouble finding children in the daytime and poor census and map accuracy led to difficulties estimating the target age group, enumerating houses, and defining EA boundaries. While not especially pronounced in these studies, non-participation rates, cost, and time can all be reasonably assumed to be higher in community TAS than in school TAS. Of note, the number of field days for school surveys was heavily skewed by the considerable time taken in the Philippines due to severe weather and poor accessibility to insecure areas in the EU. Moreover, the level of planning, training, sensitization, and field effort required for the community-based surveys in Burkina Faso and Tanzania were qualitatively much higher as reported by field staff and supervisors. Perhaps if more community-based TAS were conducted in this study and if time included the planning stage and was measured in person-hours rather than days, differences between school and community-based surveys would have been more evident. Community-based TAS are also limited by having to often sample eligible children on evenings or weekends outside of regular school hours. A more critical assessment of the 75% enrolment rate requirement for TAS school surveys could, therefore, have important implications if this threshold could be justifiably lowered. A comparison of school and community-based TAS is also important to disprove any selection bias that may occur by only sampling school children, namely that those not attending school may also not be attending MDAs and are at a higher risk for infection. Preliminary results from separate TAS studies appear to suggest there is no statistically significant difference or change in the TAS-recommended outcome for EUs with school primary enrolment rates as low as 59% [18]. Although the majority of TAS EUs are still likely to qualify for school surveys, validation of such results would greatly streamline the overall efficiency of the TAS sampling strategy if school surveys could be used on a wider or exclusive basis.

The composition of the TAS EU requires careful consideration to ensure that uniform epidemiological conditions persist across the EU. Despite the TAS being designed to provide an accurate EU-wide assessment, an EU that is smaller in area would presumably be more likely to include a self-sustaining subpopulation in its cluster sample (if such a ‘hotspot’ existed), but it might also be more cost prohibitive at a regional or national scale. In contrast, combining multiple IUs into one larger EU is more cost-effective, but clusters are spread more thinly across the EU and may miss potential hotspots where infection may persist in a focal area despite the overall EU successfully passing the TAS. A simple linear regression analysis of the EUs in this study showed moderate correlation between the cost of the TAS and EU area size, although cost is dependent on the geographical setting (e.g. transportation costs in Vanuatu were understandably greater than in Togo and Ghana despite relatively similar EU area sizes). The maximum limit of 2 million people for an EU also requires evidence; however, as the average EU population here was approximately 250,000 with a maximum of 682,000, no information about the validity of extremely large EU populations can be ascertained from this study.

Identification of cost and epidemiological appropriateness of EUs may also be aided by spatial modeling or related research to determine additional criteria that is pertinent to defining an ideal EU size or cost for TAS. Although there was no evidence of major differences between rural and non-rural clusters in our study, MDA coverage and compliance might differ considerably in both areas. Likewise, cross-border infection with high-endemic neighboring IUs or other countries may increase the risk of transmission into the TAS EU. In the Dominican Republic study, some evidence of cross-border infection from Haitian immigrants was described in bordering EAs. Other high-risk factors could persist in specific parts of an EU but not others. In the Philippines, a census evaluation of 533 TAS-eligible children was conducted in a sub-area of the EU where there is a high concentration of certain axillary plants known to support breeding of LF vectors and increase inhabitants' risk of exposure and infection. Though no positive cases or significant difference from the rest of the EU was detected in the high-risk area (unpublished data), such factors should be carefully examined and accounted for when classifying TAS EUs in order to maintain a fairly homogeneous EU so far as risk of LF infection can be assessed.

### Post-MDA Surveillance

TAS is currently recommended for EUs in post-MDA surveillance mode using an identical methodology to EUs evaluating the decision to stop or continue MDA. The results in this study support the reliability of this strategy but because TAS is not powered to detect change or designed to identify hotspots, post-MDA surveillance would best be complemented in the short and long term with other, complementary diagnostic tests and surveillance methods. In particular, antibody testing using Bm14, Bm33, or Wb123 assays may be highly suitable for post-MDA surveillance because it is more sensitive than antigen testing and may be superior to TAS for early detection of residual or resurgent LF infection. Initial findings from American Samoa and Haiti comparing filarial antigen and antibody responses seem to indicate that the antibody responses may be early markers of infection and not just exposure [19], [20]. The development of multiplex tools for NTD surveillance further facilitates the ability to conveniently examine several parameters at once [21], [22]. Xenomonitoring may also be a useful complementary post-MDA surveillance strategy because advances in molecular technology give it the potential to identify low-level LF infection in vector mosquitoes while being ‘non-invasive’ to the human population. Particularly in the majority of countries where filariasis is transmitted by *Culex* mosquitoes, efficient collection techniques exist and early results have been promising [23]–[25]. Furthermore, preliminary analysis of mosquitoes collected in American Samoa and Sri Lanka, in conjunction with these TAS studies, shows that xenomonitoring may provide comparable transmission markers and offer a cost-effective addition to the periodic post-MDA surveys where appropriately trained entomology teams are available (unpublished data). Longer term, post-TAS surveillance may also best be met through passive surveillance strategies using appropriate sentinel groups for routine blood monitoring or through malaria- or other disease-surveillance efforts [12], [22], [26].

Utilizing the antibody-based critical cutoff values for *Brugia spp.* EUs remains a concern for the current TAS protocol. While successfully passing the TAS based on more conservative thresholds increases the confidence of the results, the antibody-based thresholds may be overly restrictive, compared to the antigen-based thresholds for *W. bancrofti*. Additionally, the design effects calculated in the two *Brugia spp.* TAS (Indonesia and Malaysia) were notably higher than those assumed for calculating TAS sample sizes. In Malaysia, the large design effect can be partially attributed to a greater number of positive cases found in the EU than normally presumed by TAS. In Indonesia, however, the sample size and number of positive cases were similar to Burkina Faso yet the design effect was 2–3 times greater. Such findings may be indicative of inherent epidemiological differences of the respective EUs, but also warrant further investigation of the implications of evaluating filarial antigen and antibody using the same decision criteria.

Interruption of ongoing LF transmission and cessation of MDA in an LF endemic area are milestone achievements but ones that require careful determination and accurate assessment. TAS guidelines are currently in place for stopping MDA and post-MDA surveillance and can be carried out effectively and efficiently with recommendations and best practices identified through the operational research experiences here. While the general sampling strategy has proven to be robust and pragmatic, thresholds and sample sizes may need to be modified as new diagnostic tools become available and validated. The ability of the TAS, however, to detect recent or ongoing LF transmission in hotspots within an EU that passes the critical threshold is still untested and requires longer-term empirical evidence. Additional research into the composition of EUs and mechanisms for hotspot detection and post-MDA surveillance will only help evolve and strengthen the current guidelines. From a broader perspective, the survey design principle of the TAS can be realistically applied and adapted to other NTDs as they reach similar points in their programs. The TAS may also provide a very opportune platform and sampling strategy to integrate assessments for co-endemic NTDs such as onchocerciasis and STH. Continued deployment and refinement of the TAS, therefore, is essential not only for LF elimination programs but potentially to the wider NTD community as well.

## Supporting Information

Checklist S1 STROBE checklist.
(DOC)
